# Genetic association between *STAT4* and primary Sjögren’s syndrome in Han Chinese women

**DOI:** 10.3389/fgene.2025.1628428

**Published:** 2025-07-23

**Authors:** Fangfang Li, Junhui Lu, Chao Cen, Wanqiu Zhen, Jiaojiao Zhang, Shengming Wang

**Affiliations:** ^1^ The First Clinical College, Chongqing Medical University, Chongqing, China; ^2^ Department of Ophthalmology, The Affiliated Huai’an Hospital of Xuzhou Medical University, Huai’an, Jiangsu, China; ^3^ College of Stomatology, Chongqing Medical University, Chongqing, China; ^4^ Department of Stomatology, The Affiliated Huai’an Hospital of Xuzhou Medical University, Huai’an, Jiangsu, China

**Keywords:** genetic variants, susceptibility, primary Sjögren’s syndrome, single nucleotide polymorphism, STAT4

## Abstract

**Introduction:**

STAT4, a pivotal transcription factor governing immune and inflammatory responses, has been implicated in autoimmune pathogenesis. This case-control study aimed to examine the relationship between STAT4 single-nucleotide polymorphisms (SNPs) and primary Sjögren’s syndrome (pSS) in a female Chinese Han population, exploring potential genetic mechanisms underlying pSS susceptibility.

**Methods:**

Six STAT4 single-nucleotide polymorphisms (rs10931481, rs1400656, rs10168266, rs3821236, rs7601754, and rs10174238) were genotyped using MassARRAY, with *STAT4* expression determined by quantitative real-time PCR and cytokine levels assessed via ELISA.

**Results:**

The rs10168266-C allele emerged as a significant risk factor for pSS, with CC homozygotes exhibiting elevated disease susceptibility compared to CT/TT carriers (Pc = 0.001, OR = 1.905). Conversely, the T allele conferred protection (Pc = 0.002, OR = 0.575), and CT genotypes were underrepresented in patients (Pc = 0.003, OR = 0.539). Notably, rs10168266-CC individuals displayed elevated *STAT4* expression in peripheral blood mononuclear cells and elevated serum IL-6 levels compared to T allele carriers (both P < 0.05).

**Discussion:**

This study represents the initial investigation to uncover the genetic association between the STAT4 gene and pSS in Han Chinese women. The rs10168266 polymorphism in the STAT4 gene is a novel genetic determinant of pSS susceptibility in female Chinese Han populations. The mechanism may involve dysregulation of IL-6 signaling driven by STAT4, offering a theoretical foundation for the advancement of gene therapy.

## 1 Introduction

Primary Sjögren’s syndrome (pSS) is a prevalent chronic autoimmune disorder characterized by chronic lymphocytic infiltrations in the exocrine glands that result in symptoms such as dry eyes and dry mouth (Mariette and Criswell, 2018a; Mariette and Criswell, 2018b). In addition to these local symptoms, the disease can be manifested systemically, affecting areas such as the joints, lungs, and nervous system. Approximately 0.5%–1.5% of the global population is impacted by pSS, showing a significant female predominance with a female-to-male ratio of 9:1 (Negrini et al., 2022; Wang et al., 2021a; Alani et al., 2018). Although the onset of pSS is postulated to be the result of a complex interplay between environmental factors and genetic predisposition, the complete etiology of pSS is not fully understood. Its pathogenesis is complex and involves multiple factors such as genetics, immunology, and environmental influences (Jin et al., 2023; Brito-zerón et al., 2017; Wu et al., 2024; Mieliauskaitė et al., 2023). Therefore, the identification of new genes associated with this disease could provide a deeper insight into the mechanisms underlying the development of pSS. Improvements in our understanding would greatly enhance our exploration of the disease’s progression and potentially lead to novel therapeutic strategies and improved patient care.

A wide array of cytokines converge on a pathway that relies on Janus kinase/signal transducer and activator of transcription (JAK/STAT) signaling, which encompasses a set of intracellular messengers that facilitate cellular differentiation (Su et al., 2025; Mcgraw, 1977; Zuo et al., 2025; Bhadauriya et al., 2025; Ke et al., 2017). This pathway integrates signals from a diverse range of cytokines, acting as a crucial link between extracellular stimuli and intracellular gene expression (Lv et al., 2024). Upon cytokine binding to its receptor, the associated JAK kinases are activated, initiating a phosphorylation cascade that ultimately leads to the activation of STAT proteins. Once activated, STAT proteins translocate to the nucleus, where they regulate the transcription of genes involved in cellular differentiation, proliferation, and cytokine synthesis. Among the seven members of the STAT family, STAT4 plays a particularly critical role in orchestrating immune responses. STAT4 can be activated by cytokines such as type I IFNs, IL-12, and IL-23. Upon activation, STAT4 translocates to the nucleus, where it promotes the transcription of genes encoding cytokines like IFN-γ and TNF-α, driving the differentiation and activation of Th1 cells (Xia et al., 2023; Jin et al., 2018). Additionally, IL-23-mediated activation of STAT4 contributes to the differentiation of Th17 cells, which are key players in proinflammatory responses. As a result, STAT4 serves as a central mediator in both protective immune responses and the pathogenesis of immune-mediated diseases (Xia et al., 2023; Jin et al., 2018).

The STAT4 gene is located at the 2q32.2 locus on chromosome 2, spanning approximately 220 kilobases, encompassing 24 exons interspersed with 23 introns. The encoded STAT4 protein has a molecular mass of about 84 kDa and is composed of 748 amino acid residues. Genetic polymorphisms within the STAT4 gene have been associated with altered immune responses and increased susceptibility to several autoimmune diseases, including multiple sclerosis, rheumatoid arthritis (RA), and systemic lupus erythematosus (SLE) (Yi et al., 2013; Dieudé et al., 2009; Liu et al., 2024a; Tolomeo and Cascio, 2024; Xia et al., 2024; Sigurdsson et al., 2008). These findings highlight the potential role of STAT4 in the genetic architecture of autoimmune disorders. Given the established role of STAT4 in immune regulation and its association with other autoimmune diseases, we hypothesized that SNPs in the STAT4 gene may contribute to pSS susceptibility. PSS exhibits a striking female predominance, with reported female-to-male ratios of 9:1. This sex bias reflects both genetic and hormonal influences on disease susceptibility, making females the primary demographic for pSS research. We conducted the study in a cohort of female Chinese Han patients with pSS and age-matched healthy controls to elucidate the genetic mechanisms underlying pSS susceptibility in this population.

## 2 Materials and methods

### 2.1 Study population

PSS exhibits a striking female predominance, with reported female-to-male ratios of 9:1 (Mariette and Criswell, 2018b; Negrini et al., 2022; Wang et al., 2021a; Alani et al., 2018). This sex bias reflects both genetic and hormonal influences on disease susceptibility, making females the primary demographic for pSS research. We conducted this case–control study on 269 female patients with a diagnosis of pSS and 594 healthy women who were age matched. All patients were recruited from the Huai’an Second People’s Hospital between January 2020 and December 2024, and met the 2016 American/European consensus criteria for pSS. Exclusion criteria were recent blood transfusions (<3 months), concurrent infections, autoimmune or systemic comorbidities, and pregnancy. Diagnoses were independently confirmed by two board-certified rheumatologists. This study protocol adhered to the Declaration of Helsinki, and written informed consent was obtained from all participants.

### 2.2 SNP selection

Six tagger SNPs of STAT4 (rs10931481, rs1400656, rs10168266, rs3821236, rs7601754, and rs10174238) were selected based on a literature review and their functional relevance to autoimmune disorders. Inclusion criteria included (1) a minor allele frequency (MAF) > 5% in Han Chinese populations; (2) a linkage disequilibrium (LD) *r* ^2^ < 0.8; (3) and a Hardy–Weinberg equilibrium (HWE) P value ≥0.05 in the controls.

### 2.3 DNA isolation

Peripheral blood samples were collected in EDTA-coated vacutainers and immediately stored at −80°C. Genomic DNA was extracted using the UPure Blood DNA Extraction Plus Kit (BioKeystone Technologies^®^, China), following the manufacturer’s protocol. We quantified DNA purity and concentration spectrophotometrically (NanoPhotometer NP80 Touch, Implen GmbH, Germany), with samples meeting stringent quality criteria (A260/A280 ≥ 1.8; concentration ≥30 ng/μL). Qualified DNA was adjusted to a concentration of 30–50 ng/μL using nuclease-free water and archived at −20°C until downstream analyses.

### 2.4 Genotyping

We performed genotyping with the MassARRAY iPLEX system (Sequenom, San Diego, CA, United States). Primer pairs for target SNPs were designed using Agena Bioscience’s Assay Design Suite v2.0, with amplification and extension reactions performed following manufacturer-specified protocols. Allele discrimination was achieved through sequential steps of PCR amplification, shrimp alkaline phosphatase (SAP) treatment, single-base extension, and matrix-assisted laser desorption/ionization time-of-flight mass spectrometry (MALDI-TOF MS). To ensure analytical rigor, genotype calls were independently verified by two researchers blinded to clinical phenotypes.

### 2.5 Isolation of peripheral blood mononuclear cells (PBMCs) and real-time quantitative polymerase chain reaction (PCR)

A whole-blood sample from a newly recruited group of 60 genotyped healthy individuals was used to isolate peripheral blood mononuclear cells (PBMCs) using the Ficoll-Hypaque density-gradient centrifugation method. RNA was extracted and purified from the PBMCs using a universal RNA extraction kit (Accurate Biology, AG21017, China), and subsequently reverse transcribed into cDNA for real-time quantitative PCR using the Evo M-MLV RT Premix (Accurate Biology, AG11705, China). We analyzed the expression level of the relative target gene by applying ABI 7500 Software v2.0.6, and the outcomes were normalized with GAPDH expression as an internal control using the 2^−ΔΔCT^ method. Primers specific for *STAT4* were obtained from Sango Biotech (Shanghai, China). For the *STAT4* gene, the forward primer sequence was 5′-GCC​GGT​TGT​CAA​ATC​CCT​TAC-3′, and the reverse primer sequence was 5′-TGT​GAC​TGC​TGT​CTT​GAT​TCC​CT-3'. For the *GAPDH* gene, the forward primer sequence was 5′-CTG​CCA​ACG​TGT​CAG​TGG​TG-3′, and the reverse primer sequence was 5′-TCA​GTG​TAG​CCC​AGG​ATG​CC-3′.

### 2.6 ELISA for STAT4 and cytokines

STAT4 and various cytokines (including IFNγ, TNFα, IL-1β, IL-6, IL-12, IL-17A, and IL-23) have been reported to play a role in the development of pSS. We consequently investigated whether rs10168266 potentially and indirectly modulated the release of these cytokines. Plasma samples were isolated from whole blood collected from the same 60 genotyped healthy individuals described in Section 2.5. The levels of STAT4, IFNγ, TNFα, IL-1β, IL-6, IL-12, IL-17, and IL-23 in plasma were then determined using the ELISA method with ELISA kits ordered from DLdevelop Co., Ltd. (Wuxi, China).

### 2.7 Statistical analysis

Data were analyzed using SPSS 25.0 (IBM^®^, United States) and visualized with GraphPad Prism 7 (La Jolla^®^, United States). We examined the HWE via chi-squared tests. The patterns of linkage disequilibrium between the SNPs were compared using Haploview version 4.0 (Cambridge, MA, United States). We compared allelic frequencies in the patient and control groups using chi-squared tests. P values were corrected using the Bonferroni method by adjusting for the six tested SNPs. A statistically significant threshold of 0.05 was adopted for the study. The associations between these polymorphisms and pSS risk were statistically assessed using Pearson’s Chi-squared and Fisher’s exact-probability tests, and quantitatively evaluated with odds ratios (OR) and 95% confidence intervals (CI). We analyzed expression levels for *STAT4* and various cytokines between the two genotypic groups by implementing the nonparametric Mann–Whitney U test.

## 3 Results

### 3.1 Clinical features of pSS patients and controls

This study comprised PSS patients withaa mean age of 49.09 ± 11.46 years and healthy subjects withaa mean age of 48.13 ± 11.34 years. As detailed in Table 1, patients demonstrated predominantly glandular dysfunction, with 84.39% exhibiting severe xerostomia (unstimulated saliva flow <0.1 mL/min) and 74.72% showing reduced tear secretion (Schirmer’s test <5 mm/5 min). Serologically, the positivity rates for anti-SSA (Ro) and anti-SSB (La) antibodies were 63.57% and 47.96%, respectively, while ANA was positive in 85.13%. In addition, a range of immune-related dysfunctions were identified. Approximately one-third (33.09%) of patients exhibited hypergammaglobulinemia, while nearly 30.11% had a positive rheumatoid factor. Leukopenia was present in 21.19% of cases. Labial gland biopsies confirmed lymphocytic infiltration in 65.06% of patients, with 5.20% negative results and 29.74% of biopsies not performed. Systemic involvement included arthritis (26.77%) and Raynaud’s phenomenon (14.87%), with rare occurrences of interstitial lung disease (4.83%) and myositis (1.12%).

**TABLE 1:** Clinical symptoms and serological markers of patients with pSS.

Variable	Frequency	%
Clinical features		
Xerophthalmia (Schirmer’s test <5 mm in 5 min)	201	74.72
Rose Bengal score ≥4 (according to van Bijsterveld’s scoring system)	129	47.96
Xerostomia (Unstimulated whole saliva flow rate <0.1 mL/min)	227	84.39
Laboratory features		
Anti-SSA (Ro)	171	63.57
Anti-SSB (La)	129	47.96
ANA	229	85.13
Hypergammaglobulinemia	89	33.09
Rheumatoid factor	81	30.11
Leukopenia	57	21.19
Hypocomplementemia	29	10.78
Monoclonal component	27	10.04
Labial salivary gland biopsy		
Positive	175	65.06
Negative	14	5.20
Not performed	80	29.74
Extra-glandular manifestations		
Fever	13	4.83
Weight loss	14	5.20
Arthritis	72	26.77
Myositis	3	1.12
Vasculitis	10	3.72
Raynaud phenomenon	40	14.87
Interstitial lung disease	13	4.83

The criteria for positivity or diagnosis of each indicator are as follows: ANA, was defined as positive when the titer was ≥1:160; the positivity ofanti-SsA, anti-SsBand theumatoid factor was determined according to the cut-off values set by the reference laboratory; hypergammaglobulinemia was diagnosed when the total immunoglobulin accounted for ≥20% of the total proteins; leukopenia was diagnosed when the white blood cell count was <4,000/mm3; hypocomplementemia was diagnosed when the C3 level was <80 mg/dL and/or the C4 level was <15 mg/dL.

### 3.2 Genotypic and allelic frequencies of tested SNPs in cases and controls

The basic information on the candidate SNPs in this study is presented in Table 2. We observed no significant departure from the HWE in the six SNP loci (Table 3 summarizes the genotypic and allelic frequencies of the six SNPs of the *STAT4* gene in both pSS patients and healthy volunteers). We ascertained that of the six SNPs only rs10168266 was associated with pSS. The minor T allele of rs10168266 was related to a reduced risk of pSS compared to the major C allele [Pc = 0.002, adjusted OR = 0.575 (95% CI, 0.424–0.781)], and the CC genotype resulted in an increased risk of pSS [Pc = 0.001, OR = 1.905 (95% CI, 1.352–2.684)]. We also found that the CT genotype frequency of rs10168266 was lower in the patient group than in the controls [Pc = 0.003, OR = 0.539 (95% CI, 0.379–0.769)]. However, there was no significant association of rs10931481, rs1400656, rs3821236, rs7601754, or rs10174238 polymorphism with pSS risk.

**TABLE 2:** Basic information of SNPs in this study.

No.	SNP ID	Position	Role	Alleles	MAF		P-HWE
					Case	Control	
1	rs10931481	2:191090126	Intron Variant	G>A	0.494	0.475	0.071
2	rs1400656	2:191070307	Intron Variant	A>G	0.157	0.154	0.420
3	rs10168266	2:191071078	Intron Variant	C>T	0.108	0.178	0.244
4	rs3821236	2:191038032	Intron Variant	G>A	0.412	0.419	0.885
5	rs7601754	2:191075725	Intron Variant	A>G	0.133	0.127	0.108
6	rs10174238	2:191108308	Intron Variant	A>G	0.281	0.280	0.052

SNP, single-nucleotide polymorphisms; MAF, minor allele frequency; HWE, Hardy–Weinberg equilibrium. P < 0.05 indicates statistical significance.

**TABLE 3:** Association of six SNPs with pSS.

SNPs	Genotype/Allele	Case		Control		*P*	*P*c^a^	OR (95% CI)
		n	Frequency	n	Frequency			
rs10931481	GG	74	0.289	160	0.289	0.994	NS	0.999 (0.720, 1.385)
	AG	111	0.434	259	0.468	0.356	NS	0.869 (0.645, 1.171)
	AA	71	0.277	134	0.242	0.287	NS	1.200 (0.858, 1.679)
	G	259	0.506	579	0.524	0.509	NS	0.932 (0.756, 1.149)
	A	253	0.494	527	0.476	0.509	NS	1.073 (0.870, 1.324)
rs1400656	AA	182	0.711	394	0.706	0.888	NS	1.024 (0.739, 1.418)
	AG	68	0.266	150	0.269	0.924	NS	0.984 (0.704, 1.375)
	GG	6	0.023	14	0.025	0.888	NS	0.933 (0.354, 2.455)
	A	432	0.844	938	0.841	0.868	NS	1.025 (0.769, 1.366)
	G	80	0.156	178	0.159	0.868	NS	0.976 (0.732, 1.301)
rs10168266	CC	204	0.785	371	0.657	2.024 × 10^−4^	0.001	1.905 (1.352, 2.684)
	CT	51	0.196	176	0.312	0.001	0.003	0.539 (0.379, 0.769)
	TT	5	0.019	18	0.032	0.306	NS	0.596 (0.219, 1.623)
	C	459	0.883	918	0.812	3.565 × 10^−4^	0.002	1.738 (1.280, 2.360)
	T	61	0.117	212	0.188	3.565 × 10^−4^	0.002	0.575 (0.424, 0.781)
rs3821236	GG	85	0.332	188	0.332	0.99	NS	1.002 (0.732, 1.371)
	AG	124	0.484	274	0.483	0.976	NS	1.005 (0.748, 1.350)
	AA	47	0.184	105	0.185	0.957	NS	0.989 (0.676, 1.448)
	G	294	0.574	650	0.573	0.969	NS	1.004 (0.813, 1.240)
	A	218	0.426	484	0.427	0.969	NS	0.996 (0.806, 1.230)
rs7601754	AA	194	0.758	428	0.754	0.895	NS	1.024 (0.726, 1.443)
	AG	56	0.219	125	0.22	0.966	NS	0.992 (0.695, 1.418)
	GG	6	0.023	15	0.026	0.802	NS	0.885 (0.339, 2.307)
	A	444	0.867	981	0.864	0.842	NS	1.032 (0.759, 1.401)
	G	68	0.133	155	0.136	0.842	NS	0.969 (0.714, 1.317)
rs10174238	AA	123	0.479	273	0.48	0.975	NS	0.995 (0.741, 1.337)
	AG	115	0.447	253	0.445	0.94	NS	1.012 (0.752, 1.360)
	GG	19	0.074	43	0.076	0.934	NS	0.977 (0.557, 1.712)
	A	361	0.702	799	0.702	0.993	NS	1.001 (0.797, 1.257)
	G	153	0.298	339	0.298	0.993	NS	0.999 (0.795, 1.254)

Abbreviation: OR, odds ratio; CI, confidence interval; NS, no significance.

^a^ *P* values were adjusted with the Bonferroni method by multiplying the number of candidate SNPs by 6.

### 3.3 Associations between tested polymorphisms and disease phenotypes

We investigated the relationships between six genetic polymorphisms and disease phenotypes. The rs10168266 polymorphism exhibited significant associations with xerophthalmia and ANA status (Table 4). The rs10168266-CC genotype was notably more prevalent among patients with xerophthalmia [P = 0.023, OR = 2.105 (95% CI: 1.097–4.042)], indicating an increased risk of developing the condition. Upon examining alleles, the C allele was also more common in patients with xerophthalmia [P = 0.02, OR = 1.959 (95% CI: 1.104–3.478)]. Regarding ANA status, the rs10168266-CC genotype was more frequent in ANA-positive patients [P = 0.044, OR = 2.249 (95% CI: 1.006–5.029)], suggesting a higher risk of ANA positivity. The C allele of rs10168266 also showed a trend towards increased frequency in ANA-positive patients (P = 0.047), although the lower limit of the 95% CI for the OR (0.999–4.019) was close to 1, which hindered achieving strict statistical significance. In contrast to rs10168266, we found no associations between the other tested SNPs and the disease phenotypes being studied (Supplementary Table S1).

**TABLE 4:** Associations between disease phenotypes and rs10168266.

Phenotypes	Genotype/Allele	Positive		Negative		*P*	OR (95% CI)
		n	Frequency	n	Frequency		
Xerophthalmia	CC	164	0.816	40	0.678	0.023	2.105 (1.097,4.042)
	CT	34	0.169	17	0.288	0.043	0.503 (0.257,0.986)
	TT	3	0.015	2	0.034	0.351	0.432 (0.07,2.647)
	C	362	0.9	97	0.822	0.02	1.959 (1.104,3.478)
	T	40	0.1	21	0.178	0.02	0.51 (0.288,0.906)
ANA	CC	184	0.803	20	0.645	0.044	2.249 (1.006,5.029)
	CT	41	0.179	10	0.323	0.059	0.458 (0.201,1.045)
	TT	4	0.017	1	0.032	0.574	0.533 (0.058,4.931)
	C	409	0.893	50	0.806	0.047	2.003 (0.999,4.019)
	T	49	0.107	12	0.194	0.047	0.499 (0.249,1.001)

### 3.4 Effect of positive SNPs on STAT4 gene expression

**FIGURE 1:**
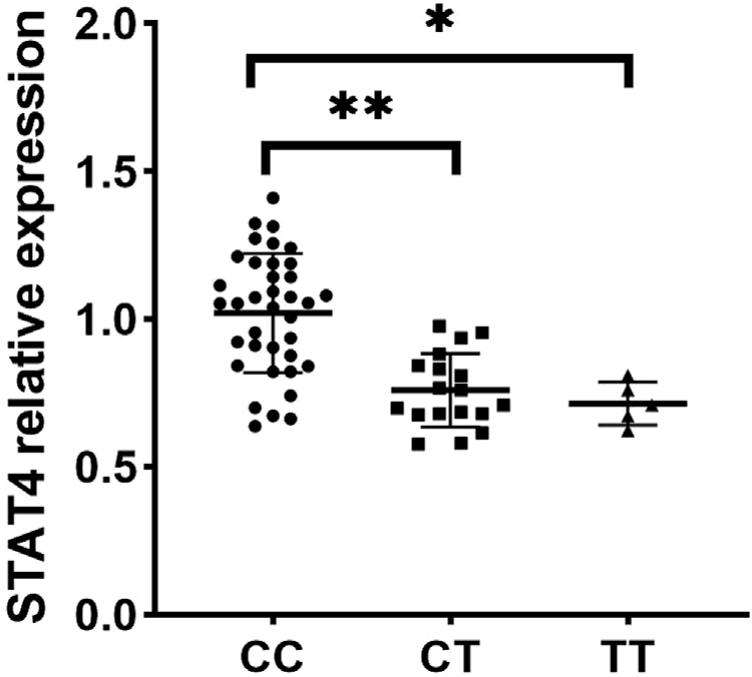
Expression analysis of *STAT4* between various genotypes of rs10168266 (CC=37, CT=18, TT=5) in PBMCs from healthy controls. Data are presented as mean±SEM. Dot plot showing STAT4 relative expression across three genotypes: CC, CT, and TT. The CC group has the highest expression, followed by CT and TT. Asterisks indicate statistically significant differences between groups (p-values), with one asterisk for CC vs. TT (P<0.01) and two asterisks for CC vs. CT (P<0.001).

Since rs10168266 exhibited a correlation with pSS, we included it in the subsequent functional study. To investigate the function of rs10168266, we adopted RT-qPCR to quantitatively determine the expression of target genes in the PBMCs obtained from a newly recruited group of 60 genotyped healthy individuals. Our results showed that *STAT4* expression level in PBMCs was higher in rs10168266 CC carriers relative to CT and TT carriers (both *P* < 0.05, Figure 1).

### 3.5 Effects of SNPs on STAT4 and cytokine production

**FIGURE 2:**
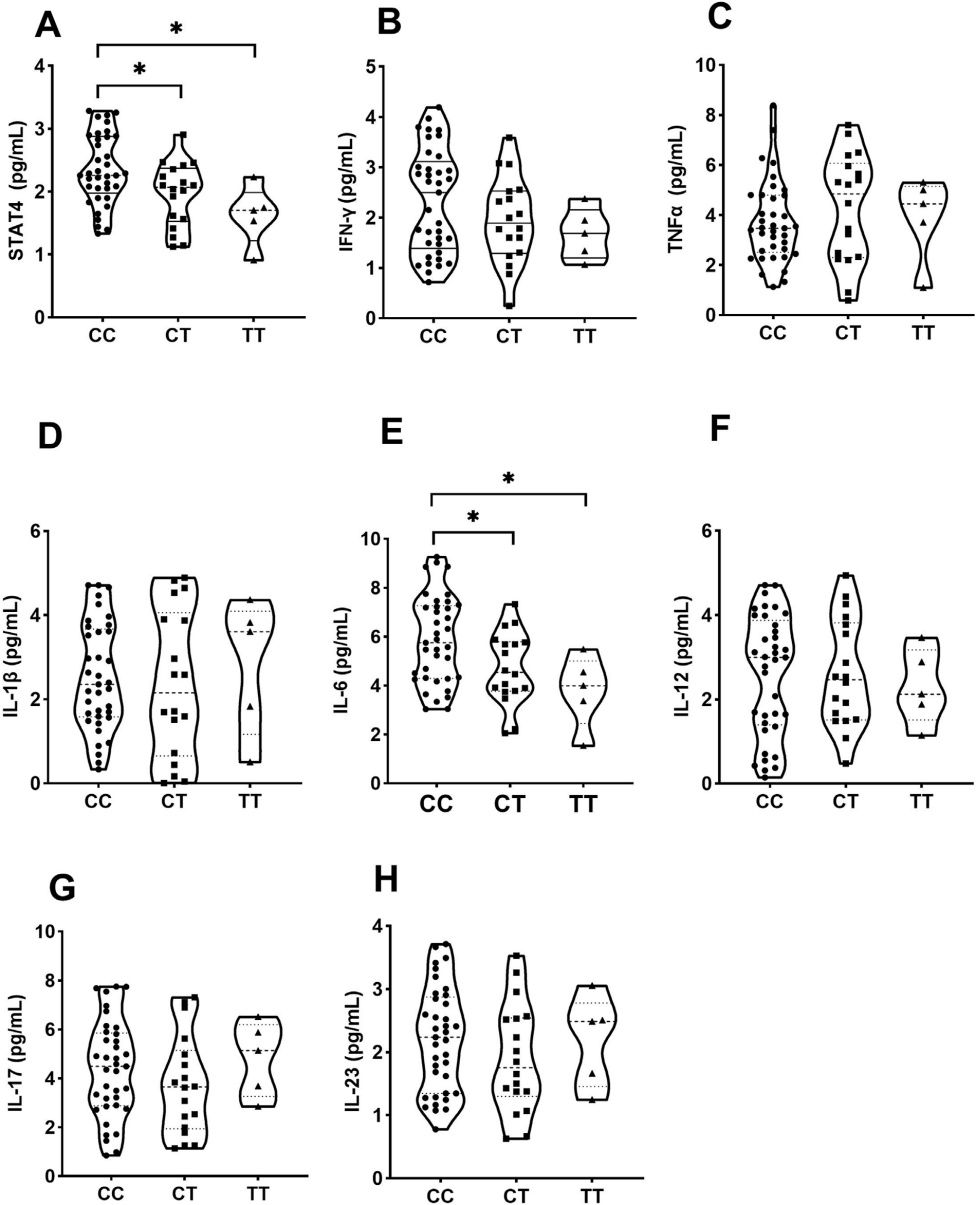
Influence of various genotypes of rs10168266 (CC=37, CT=18, TT=5) on STAT4 and cytokine production. Data are presented as mean±SD. Violin plots display various cytokine levels in picograms per milliliter for genotypes CC, CT, and TT. Panels A to H show STAT4, IFN-γ, TNFα, IL-1β, IL-6, IL-12, IL-17, and IL-23, respectively. Statistical significance is indicated with asterisks in panels A and E.

Individuals carrying the CT/TT genotype of rs10168266 displayed lower levels of serum STAT4 than CC homozygotes (*P* = 0.031, *P* = 0.011, Figure 2A). In the comparison between groups, a significantly higher level of IL6 was also observed in individuals with the rs10168266-CC genotype relative to with rs10168266-CT/TT (*P* = 0.037, *P* = 0.033, Figure 2E). We discerned no association between *STAT4* gene polymorphism and production of any other cytokine (Figures 2 B, C, D, F, G, H).

## 4 Discussion

As a pivotal transcription factor that governs both innate and adaptive immunity, STAT4 orchestrates immune homeostasis via its dual roles in signal transduction and gene regulation. Our investigation into six *STAT4* polymorphisms (rs10931481, rs1400656, rs10168266, rs3821236, rs7601754, and rs10174238) identified rs10168266 as a novel susceptibility locus for pSS, with the C-allele conferring elevated disease risk. This finding corroborates emerging evidence that implicates *STAT4* variants in autoimmune dysregulation, but also highlights distinct genetic architecture across pathologies. Our data underscored rs10168266 as a pSS-specific risk marker, and potentially reflects disease-specific etiological pathways or population-specific linkage disequilibrium patterns. Notably, the rs10168266-C allele demonstrated a dosage-dependent effect on serum STAT4 and IL-6 levels in healthy carriers, suggesting a functional interplay between genetic variation and pro-inflammatory cytokine production that may drive glandular inflammation in pSS (Negrini et al., 2022; Hou et al., 2024).

Our research findings align with those of several previous studies. A study by Japanese researchers involving 308 SLE patients and 306 control subjects found that rs10168266 was associated with an increased risk of SLE. Furthermore, it was found that the association between these polymorphisms and SLE was more pronounced in the Japanese population than in European or American populations (Kawasaki et al., 2008). An analysis conducted by Jia-Min Wang and their co-authors aimed to determine the associations between *STAT4* polymorphisms and SLE risk, and they found that the TT genotypes of rs10168266 were associated with SLE (Wang et al., 2021b). A meta-analysis suggested that rs10168266 was significantly associated with the risk of primary biliary cholangitis (Zhang et al., 2018), as well as with the risk of HBV infection, and it can serve as predictor of PEG-IFN-alpha treatment outcomes (Lei et al., 2024). While rs10168266 has been linked to other autoimmune diseases, its specific role in pSS susceptibility—particularly within the Han Chinese female population—represents a novel finding.

The mechanistic relevance of rs10168266 warrants examination despite its non-coding localization. Positioned within an intron, this SNP may influence transcriptional regulation through long-range chromatin interactions or by tagging functional variants in tightly linked regions. Previous studies have identified rs10168266 as part of an extended haplotype that encompasses rs7582694, rs7568275, and rs7574865—a locus associated with augmented *STAT4* expression and hyperresponsiveness to IL-12/IFN-⍺ signaling in SLE and RA (Namjou et al., 2009). Our observation of heightened STAT4 and IL-6 levels in individuals harboring rs10168266-CC parallels findings by Lamana et al., who reported allele-dependent *STAT4* overexpression in immune cells, potentially amplifying Th1/Th17 polarization (Lamana et al., 2012; Lamana et al., 2015). Such dysregulation could disrupt salivary gland homeostasis by promoting autoreactive lymphocyte infiltration and sustaining inflammatory cascades, both hallmarks of pSS pathogenesis (Negrini et al., 2022; Wu et al., 2024; Mieliauskaitė et al., 2023; Hou et al., 2024). Furthermore, IL-6 synergizes with STAT4 to reinforce Th17 differentiation while inhibiting regulatory T-cell function, thus creating a self-perpetuating inflammatory milieu (Zhang et al., 2020). These data collectively position rs10168266 as a modulator of STAT4-driven immune hyperactivity, although cis-regulatory effects versus trans-acting epistatic interactions require further clarification through chromatin conformation assays or CRISPR-based editing. Our research findings provide new insights for the treatment of PSS. Firstly, IL-6 receptor antagonists, such as tocilizumab, present a logical approach, considering the increased serum IL-6 levels seen in individuals with the rs10168266-CC genotype. A randomized, double-blind, parallel, placebo-controlled trial designed to assess the efficacy of tocilizumab in pSS has started and the results are eagerly awaited (He et al., 2024). Secondly, direct inhibition of STAT4 signaling, through small-molecule inhibitors that target STAT4 phosphorylation or nuclear translocation, emerges as a novel therapeutic direction. Preclinical studies indicate that STAT4 deficiency diminishes autoantibody production and glomerulonephritis in a lupus mouse model (Xu et al., 2006), corroborating our observations of STAT4-IL-6 interaction in pSS. Together, these strategies exploit the mechanistic connection between rs10168266 and immune dysregulation, offering a foundation for the development of personalized treatments that address STAT4-driven inflammatory pathways in pSS.

In this study, we identified rs10168266 in STAT4 as a novel genetic determinant for pSS susceptibility in female Han Chinese, highlighting STAT4-mediated IL-6 signaling dysregulation in pSS pathogenesis. Comparing mechanisms across related autoimmune diseases reveals both shared and distinct features. SLE involves genetic, epigenetic, and environmental factors (Xu et al., 2025; Qiu et al., 2025; Martinez and Kaplan, 2025), with STAT4 regulating immune activation and IL-6 driving B-cell hyperactivity (Liu et al., 2024a; Guerrero et al., 2023), parallels to pSS, though SLE is defined by systemic autoantibody deposition (Huang et al., 2025; Liu et al., 2024b). RA, linked to MHC shared epitopes and smoking-induced citrullination (Khalid, 2024; Alm et al., 2025; Romero-castillo et al., 2024), involves STAT4 in Th1/Th17 activation and IL-6 in joint destruction (Srivastava and Rasool, 2024; Yanagisawa et al., 2022; Bravo-villagra et al., 2024). Unlike pSS, its STAT4-IL-6 polymorphism associations are less defined. IBD arises from dysregulated microbiota-immune interactions (Diez-martin et al., 2024; Somineni et al., 2021), with STAT4 promoting Th1/Th17 responses. While IL-6 is involved, its STAT4-polymorphism links are weaker than in pSS, and gut-specific inflammation distinguishes it (Liu et al., 2015). PSS features fibrosis driven by Th2/STAT4-mediated TGF-β signaling. IL-6 plays a role, but its STAT4-polymorphism associations are less clear than in pSS, with fibrosis contrasting pSS’s exocrine gland focus. In summary, these diseases share genetic predisposition, environmental triggers, and immune dysregulation but differ in specific loci, pathways, and target organs. Our rs10168266 finding adds to understanding of autoimmune pathogenesis, with future studies needed to explore analogous STAT4 mechanisms across conditions.

Five additional STAT4 SNPs tested in this study—rs10931481, rs1400656, rs3821236, rs7601754, and rs10174238—have previously been linked to susceptibility in other diseases. For example, rs10931481 is associated with increased risk of polyarticular juvenile idiopathic arthritis, and has been linked to levothyroxine sodium treatment and self-reported hypothyroidism or myxoedema (Zhu et al., 2023). The GA genotype of rs1400656 confers protective effects against lung cancer (Ma et al., 2020), while rs3821236 acts additively to elevate SLE risk (Abelson et al., 2009), and is associated with type 2 diabetes (Cui et al., 2021). Rs7601754 has been shown to increase the odds of multiple sclerosis (Bruzaite et al., 2024), and rs10174238 was identified by German researchers as protective against Crohn’s disease (Xia et al., 2024). Notably, our study found no association between these five SNPs and pSS susceptibility in the Chinese Han female cohort. The lack of replication for these established autoimmune risk variants highlights genetic heterogeneity across autoimmune phenotypes. This divergence may partially stem from population stratification, as allelic frequencies and linkage disequilibrium patterns of STAT4 variants differ significantly across ethnic groups. For instance, the rs7574865-T allele demonstrates stronger effects in European cohorts compared to East Asian populations, where alternative loci like rs10168266 emerge as primary drivers of risk (Glas et al., 2010; Shancui-Zheng et al., 2022; Chai et al., 2014). Such ethnic-specific genetic architecture underscores the critical role of population-specific studies in unraveling disease susceptibility. Additionally, pSS-specific epigenetic modifications or gene-environment interactions may influence the penetrance of risk alleles not captured in broader autoimmune analyses. By focusing on a genetically homogeneous Chinese Han population, this study minimizes confounding from LD heterogeneity, enhancing the specificity of detected associations. This approach highlights the potential for identifying disease-specific genetic factors, such as rs10168266, which may inform personalized medicine strategies for pSS. The absence of associations with SNPs implicated in other autoimmune conditions further underscores the distinct pathogenesis of pSS. Autoimmune diseases exhibit profound clinical and genetic diversity, even within the same ethnic group, reflecting the complex interplay of genetic, epigenetic, and environmental factors. Addressing this complexity requires large-scale, multi-ethnic studies with robust statistical power to distinguish shared versus disease-specific risk mechanisms. Such efforts will be essential for comprehensively mapping the genetic landscape of pSS and advancing targeted therapeutic development.

Several limitations temper the interpretation of our findings. A key limitation of this study is the exclusive enrollment of Han Chinese females, which, while minimizing confounding from population stratification and sex-specific biases, restricts generalizability to other demographic groups. The genetic basis of autoimmune disorders like pSS exhibits pronounced population heterogeneity, and STAT4 variants may exert distinct effects across diverse ethnic cohorts. For instance, replication in multi-ethnic populations—including males, other Asian subgroups, and individuals of European, African, or Hispanic ancestry—is critical to validate the global relevance of rs10168266. Such cross-population studies are essential to discern whether the observed association is specific to Han Chinese females or indicative of a broader role for STAT4 in pSS pathogenesis, particularly given the high autoimmune disease prevalence in certain ethnic groups (e.g., European or African populations). This limitation underscores the need for future investigations in diverse cohorts to establish the generalizability of these findings and clarify the ethnic-specificity of STAT4-driven immune dysregulation in pSS. A further limitation of this study lies in the use of tagger SNPs designed to capture major STAT4 haplotypes, which may have overlooked rare or structural variants with functional significance. Genome-wide approaches integrating fine-mapping and expression quantitative trait locus (eQTL) analyses could help identify causal variants and their target genes. Future studies employing whole-exome or whole-genome sequencing are needed to comprehensively assess both common and rare variants in STAT4, as this approach may uncover additional functional polymorphisms contributing to pSS susceptibility. Such investigations would bridge the gap between haplotype-based genotyping and comprehensive genomic coverage, particularly given the complex regulatory landscape of STAT4. Finally, the correlative nature of our cytokine data necessitates functional validation through *in vitro* models such as CRISPR-edited cell lines or patient-derived immune cells, so as to dissect allele-specific effects on STAT4 signaling dynamics.

In conclusion, we herein identified rs10168266 as a putative genetic determinant of pSS susceptibility that potentially operated through STAT4-mediated amplification of Th1/Th17 responses and IL-6 overproduction. While reinforcing STAT4’s centrality in autoimmune pathogenesis, our results underscored the complexity of translating genetic associations into mechanistic insights. Future investigations combining multi-ethnic cohorts, advanced genomic tools, and mechanistic studies will be critical to unraveling the molecular cascades linking *STAT4* polymorphisms to glandular dysfunction, a prerequisite for developing targeted therapies in pSS.

## Data Availability

The original contributions presented in the study are included in the article/Supplementary Material, further inquiries can be directed to the corresponding author.
