# Supplementary material for: Genetic association between STAT4 and primary Sjögren’s syndrome in Han Chinese women
Source: Front Genet. 2025 Jul 23;16:1628428. doi: 10.3389/fgene.2025.1628428 (PMC12326203; doi:10.3389/fgene.2025.1628428)
Supplement: Supplementary file 1 [file Table1.docx]

Table S1. Associations between disease phenotypes and studied polymorphisms

| **Phenotypes** | **SNPs** | **Genotype/Allele** | **+** | | **-** | | **P** | **OR (95% CI)** |
| --- | --- | --- | --- | --- | --- | --- | --- | --- |
|  |  |  | **n** | **Frequency** | **n** | **Frequency** |  |  |
| Xerophthalmia | rs10931481 | GG | 57 | 0.284 | 17 | 0.309 | 0.712 | 0.885(0.462,1.693) |
|  |  | AG | 85 | 0.423 | 26 | 0.473 | 0.509 | 0.817(0.449,1.487) |
|  |  | AA | 59 | 0.294 | 12 | 0.218 | 0.269 | 1.489(0.733,3.023) |
|  |  | G | 199 | 0.495 | 60 | 0.545 | 0.349 | 0.817(0.535,1.247) |
|  |  | A | 203 | 0.505 | 50 | 0.455 | 0.349 | 1.224(0.802,1.869) |
|  | rs1400656 | AA | 138 | 0.687 | 44 | 0.8 | 0.1 | 0.548(0.265,1.13) |
|  |  | AG | 59 | 0.294 | 9 | 0.164 | 0.053 | 2.124(0.977,4.615) |
|  |  | GG | 4 | 0.02 | 2 | 0.036 | 0.475 | 0.538(0.096,3.018) |
|  |  | A | 335 | 0.833 | 97 | 0.882 | 0.215 | 0.67(0.355,1.265) |
|  |  | G | 67 | 0.167 | 13 | 0.118 | 0.215 | 1.492(0.79,2.818) |
|  | rs10168266 | CC | 164 | 0.816 | 40 | 0.678 | 0.023 | 2.105(1.097,4.042) |
|  |  | CT | 34 | 0.169 | 17 | 0.288 | 0.043 | 0.503(0.257,0.986) |
|  |  | TT | 3 | 0.015 | 2 | 0.034 | 0.351 | 0.432(0.07,2.647) |
|  |  | C | 362 | 0.9 | 97 | 0.822 | 0.02 | 1.959(1.104,3.478) |
|  |  | T | 40 | 0.1 | 21 | 0.178 | 0.02 | 0.51(0.288,0.906) |
|  | rs3821236 | GG | 66 | 0.328 | 19 | 0.345 | 0.811 | 0.926(0.494,1.737) |
|  |  | AG | 94 | 0.468 | 30 | 0.545 | 0.306 | 0.732(0.402,1.332) |
|  |  | AA | 41 | 0.204 | 6 | 0.109 | 0.107 | 2.093(0.839,5.223) |
|  |  | G | 226 | 0.562 | 68 | 0.618 | 0.293 | 0.793(0.515,1.222) |
|  |  | A | 176 | 0.438 | 42 | 0.382 | 0.293 | 1.261(0.818,1.942) |
|  | rs7601754 | AA | 153 | 0.761 | 41 | 0.745 | 0.809 | 1.088(0.547,2.165) |
|  |  | AG | 43 | 0.214 | 13 | 0.236 | 0.721 | 0.879(0.433,1.784) |
|  |  | GG | 5 | 0.025 | 1 | 0.018 | 0.771 | 1.378(0.158,12.042) |
|  |  | A | 349 | 0.868 | 95 | 0.864 | 0.901 | 1.04(0.561,1.926) |
|  |  | G | 53 | 0.132 | 15 | 0.136 | 0.901 | 0.962(0.519,1.782) |
|  | rs10174238 | AA | 91 | 0.453 | 32 | 0.571 | 0.116 | 0.62(0.341,1.128) |
|  |  | AG | 94 | 0.468 | 21 | 0.375 | 0.217 | 1.464(0.797,2.689) |
|  |  | GG | 16 | 0.08 | 3 | 0.054 | 0.51 | 1.528(0.429,5.443) |
|  |  | A | 276 | 0.687 | 85 | 0.759 | 0.139 | 0.696(0.43,1.126) |
|  |  | G | 126 | 0.313 | 27 | 0.241 | 0.139 | 1.437(0.888,2.326) |
| Xerostomia | rs10931481 | GG | 65 | 0.286 | 9 | 0.31 | 0.788 | 0.892(0.386,2.061) |
|  |  | AG | 101 | 0.445 | 10 | 0.345 | 0.306 | 1.523(0.678,3.421) |
|  |  | AA | 61 | 0.269 | 10 | 0.345 | 0.389 | 0.698(0.307,1.585) |
|  |  | G | 231 | 0.509 | 28 | 0.483 | 0.709 | 1.11(0.642,1.918) |
|  |  | A | 223 | 0.491 | 30 | 0.517 | 0.709 | 0.901(0.521,1.557) |
|  | rs1400656 | AA | 162 | 0.714 | 20 | 0.69 | 0.788 | 1.122(0.485,2.592) |
|  |  | AG | 60 | 0.264 | 8 | 0.276 | 0.895 | 0.943(0.397,2.242) |
|  |  | GG | 5 | 0.022 | 1 | 0.034 | 0.676 | 0.631(0.071,5.594) |
|  |  | A | 384 | 0.846 | 48 | 0.828 | 0.719 | 1.143(0.552,2.365) |
|  |  | G | 70 | 0.154 | 10 | 0.172 | 0.719 | 0.875(0.423,1.811) |
|  | rs10168266 | CC | 177 | 0.78 | 27 | 0.818 | 0.616 | 0.787(0.308,2.011) |
|  |  | CT | 47 | 0.207 | 4 | 0.121 | 0.246 | 1.893(0.634,5.651) |
|  |  | TT | 3 | 0.013 | 2 | 0.061 | 0.064 | 0.208(0.033,1.292) |
|  |  | C | 401 | 0.883 | 58 | 0.879 | 0.916 | 1.044(0.472,2.306) |
|  |  | T | 53 | 0.117 | 8 | 0.121 | 0.916 | 0.958(0.434,2.117) |
|  | rs3821236 | GG | 79 | 0.348 | 6 | 0.207 | 0.129 | 2.046(0.8,5.233) |
|  |  | AG | 109 | 0.48 | 15 | 0.517 | 0.707 | 0.862(0.398,1.869) |
|  |  | AA | 39 | 0.172 | 8 | 0.276 | 0.173 | 0.545(0.225,1.319) |
|  |  | G | 267 | 0.588 | 27 | 0.466 | 0.075 | 1.639(0.947,2.838) |
|  |  | A | 187 | 0.412 | 31 | 0.534 | 0.075 | 0.61(0.352,1.056) |
|  | rs7601754 | AA | 170 | 0.749 | 24 | 0.828 | 0.352 | 0.621(0.227,1.704) |
|  |  | AG | 52 | 0.229 | 4 | 0.138 | 0.264 | 1.857(0.618,5.579) |
|  |  | GG | 5 | 0.022 | 1 | 0.034 | 0.676 | 0.631(0.071,5.594) |
|  |  | A | 392 | 0.863 | 52 | 0.897 | 0.484 | 0.73(0.301,1.77) |
|  |  | G | 62 | 0.137 | 6 | 0.103 | 0.484 | 1.371(0.565,3.326) |
|  | rs10174238 | AA | 108 | 0.476 | 15 | 0.5 | 0.803 | 0.908(0.424,1.944) |
|  |  | AG | 103 | 0.454 | 12 | 0.4 | 0.578 | 1.246(0.574,2.707) |
|  |  | GG | 16 | 0.07 | 3 | 0.1 | 0.561 | 0.682(0.187,2.496) |
|  |  | A | 319 | 0.703 | 42 | 0.7 | 0.966 | 1.013(0.563,1.823) |
|  |  | G | 135 | 0.297 | 18 | 0.3 | 0.966 | 0.987(0.549,1.777) |
| Anti-SSA (Ro) | rs10931481 | GG | 48 | 0.281 | 26 | 0.306 | 0.676 | 0.886(0.501,1.565) |
|  |  | AG | 76 | 0.444 | 35 | 0.412 | 0.619 | 1.143(0.675,1.936) |
|  |  | AA | 47 | 0.275 | 24 | 0.282 | 0.9 | 0.963(0.54,1.72) |
|  |  | G | 172 | 0.503 | 87 | 0.512 | 0.851 | 0.965(0.668,1.395) |
|  |  | A | 170 | 0.497 | 83 | 0.488 | 0.851 | 1.036(0.717,1.497) |
|  | rs1400656 | AA | 117 | 0.684 | 65 | 0.765 | 0.181 | 0.667(0.367,1.21) |
|  |  | AG | 51 | 0.298 | 17 | 0.2 | 0.094 | 1.7(0.911,3.174) |
|  |  | GG | 3 | 0.018 | 3 | 0.035 | 0.377 | 0.488(0.096,2.471) |
|  |  | A | 285 | 0.833 | 147 | 0.865 | 0.357 | 0.782(0.463,1.32) |
|  |  | G | 57 | 0.167 | 23 | 0.135 | 0.357 | 1.278(0.757,2.158) |
|  | rs10168266 | CC | 138 | 0.807 | 66 | 0.742 | 0.223 | 1.457(0.794,2.676) |
|  |  | CT | 30 | 0.175 | 21 | 0.236 | 0.244 | 0.689(0.368,1.291) |
|  |  | TT | 3 | 0.018 | 2 | 0.022 | 0.784 | 0.777(0.127,4.736) |
|  |  | C | 306 | 0.895 | 153 | 0.86 | 0.237 | 1.389(0.805,2.397) |
|  |  | T | 36 | 0.105 | 25 | 0.14 | 0.237 | 0.72(0.417,1.243) |
|  | rs3821236 | GG | 61 | 0.357 | 24 | 0.282 | 0.234 | 1.409(0.8,2.484) |
|  |  | AG | 79 | 0.462 | 45 | 0.529 | 0.309 | 0.763(0.453,1.286) |
|  |  | AA | 31 | 0.181 | 16 | 0.188 | 0.892 | 0.955(0.489,1.864) |
|  |  | G | 201 | 0.588 | 93 | 0.547 | 0.381 | 1.18(0.815,1.71) |
|  |  | A | 141 | 0.412 | 77 | 0.453 | 0.381 | 0.847(0.585,1.228) |
|  | rs7601754 | AA | 125 | 0.731 | 69 | 0.812 | 0.155 | 0.63(0.332,1.195) |
|  |  | AG | 41 | 0.24 | 15 | 0.176 | 0.249 | 1.472(0.761,2.845) |
|  |  | GG | 5 | 0.029 | 1 | 0.012 | 0.384 | 2.53(0.291,22.005) |
|  |  | A | 291 | 0.851 | 153 | 0.9 | 0.123 | 0.634(0.354,1.135) |
|  |  | G | 51 | 0.149 | 17 | 0.1 | 0.123 | 1.577(0.881,2.825) |
|  | rs10174238 | AA | 80 | 0.468 | 43 | 0.5 | 0.626 | 0.879(0.523,1.477) |
|  |  | AG | 75 | 0.439 | 40 | 0.465 | 0.687 | 0.898(0.534,1.512) |
|  |  | GG | 16 | 0.094 | 3 | 0.035 | 0.09 | 2.856(0.809,10.084) |
|  |  | A | 235 | 0.687 | 126 | 0.733 | 0.288 | 0.802(0.533,1.205) |
|  |  | G | 107 | 0.313 | 46 | 0.267 | 0.288 | 1.247(0.83,1.875) |
| Anti-SSB (La) | rs10931481 | GG | 39 | 0.302 | 35 | 0.276 | 0.637 | 1.139(0.663,1.957) |
|  |  | AG | 55 | 0.426 | 56 | 0.441 | 0.814 | 0.942(0.575,1.545) |
|  |  | AA | 35 | 0.271 | 36 | 0.283 | 0.828 | 0.941(0.544,1.627) |
|  |  | G | 133 | 0.516 | 126 | 0.496 | 0.66 | 1.081(0.764,1.529) |
|  |  | A | 125 | 0.484 | 128 | 0.504 | 0.66 | 0.925(0.654,1.308) |
|  | rs1400656 | AA | 117 | 0.684 | 65 | 0.765 | 0.181 | 0.667(0.367,1.21) |
|  |  | AG | 51 | 0.298 | 17 | 0.2 | 0.094 | 1.7(0.911,3.174) |
|  |  | GG | 3 | 0.018 | 3 | 0.035 | 0.377 | 0.488(0.096,2.471) |
|  |  | A | 285 | 0.833 | 147 | 0.865 | 0.357 | 0.782(0.463,1.32) |
|  |  | G | 57 | 0.167 | 23 | 0.135 | 0.357 | 1.278(0.757,2.158) |
|  | rs10168266 | CC | 138 | 0.807 | 66 | 0.742 | 0.223 | 1.457(0.794,2.676) |
|  |  | CT | 30 | 0.175 | 21 | 0.236 | 0.244 | 0.689(0.368,1.291) |
|  |  | TT | 3 | 0.018 | 2 | 0.022 | 0.784 | 0.777(0.127,4.736) |
|  |  | C | 306 | 0.895 | 153 | 0.86 | 0.237 | 1.389(0.805,2.397) |
|  |  | T | 36 | 0.105 | 25 | 0.14 | 0.237 | 0.72(0.417,1.243) |
|  | rs3821236 | GG | 61 | 0.357 | 24 | 0.282 | 0.234 | 1.409(0.8,2.484) |
|  |  | AG | 79 | 0.462 | 45 | 0.529 | 0.309 | 0.763(0.453,1.286) |
|  |  | AA | 31 | 0.181 | 16 | 0.188 | 0.892 | 0.955(0.489,1.864) |
|  |  | G | 201 | 0.588 | 93 | 0.547 | 0.381 | 1.18(0.815,1.71) |
|  |  | A | 141 | 0.412 | 77 | 0.453 | 0.381 | 0.847(0.585,1.228) |
|  | rs7601754 | AA | 125 | 0.731 | 69 | 0.812 | 0.155 | 0.63(0.332,1.195) |
|  |  | AG | 41 | 0.24 | 15 | 0.176 | 0.249 | 1.472(0.761,2.845) |
|  |  | GG | 5 | 0.029 | 1 | 0.012 | 0.384 | 2.53(0.291,22.005) |
|  |  | A | 291 | 0.851 | 153 | 0.9 | 0.123 | 0.634(0.354,1.135) |
|  |  | G | 51 | 0.149 | 17 | 0.1 | 0.123 | 1.577(0.881,2.825) |
|  | rs10174238 | AA | 83 | 0.485 | 40 | 0.465 | 0.759 | 1.085(0.645,1.823) |
|  |  | AG | 75 | 0.439 | 40 | 0.465 | 0.687 | 0.898(0.534,1.512) |
|  |  | GG | 13 | 0.076 | 6 | 0.07 | 0.856 | 1.097(0.402,2.994) |
|  |  | A | 241 | 0.705 | 120 | 0.698 | 0.87 | 1.034(0.693,1.542) |
|  |  | G | 101 | 0.295 | 52 | 0.302 | 0.87 | 0.967(0.648,1.443) |
| ANA | rs10931481 | GG | 68 | 0.297 | 6 | 0.222 | 0.418 | 1.478(0.571,3.824) |
|  |  | AG | 95 | 0.415 | 16 | 0.593 | 0.078 | 0.487(0.217,1.097) |
|  |  | AA | 66 | 0.288 | 5 | 0.185 | 0.258 | 1.782(0.647,4.903) |
|  |  | G | 231 | 0.504 | 28 | 0.519 | 0.844 | 0.945(0.537,1.661) |
|  |  | A | 227 | 0.496 | 26 | 0.481 | 0.844 | 1.058(0.602,1.861) |
|  | rs1400656 | AA | 162 | 0.707 | 20 | 0.741 | 0.718 | 0.846(0.342,2.095) |
|  |  | AG | 62 | 0.271 | 6 | 0.222 | 0.589 | 1.299(0.501,3.37) |
|  |  | GG | 5 | 0.022 | 1 | 0.037 | 0.621 | 0.58(0.065,5.16) |
|  |  | A | 386 | 0.843 | 46 | 0.852 | 0.862 | 0.932(0.422,2.058) |
|  |  | G | 72 | 0.157 | 8 | 0.148 | 0.862 | 1.073(0.486,2.368) |
|  | rs10168266 | CC | 184 | 0.803 | 20 | 0.645 | 0.044 | 2.249(1.006,5.029) |
|  |  | CT | 41 | 0.179 | 10 | 0.323 | 0.059 | 0.458(0.201,1.045) |
|  |  | TT | 4 | 0.017 | 1 | 0.032 | 0.574 | 0.533(0.058,4.931) |
|  |  | C | 409 | 0.893 | 50 | 0.806 | 0.047 | 2.003(0.999,4.019) |
|  |  | T | 49 | 0.107 | 12 | 0.194 | 0.047 | 0.499(0.249,1.001) |
|  | rs3821236 | GG | 75 | 0.328 | 10 | 0.37 | 0.655 | 0.828(0.362,1.896) |
|  |  | AG | 110 | 0.48 | 14 | 0.519 | 0.707 | 0.858(0.386,1.907) |
|  |  | AA | 44 | 0.192 | 3 | 0.111 | 0.304 | 1.903(0.548,6.604) |
|  |  | G | 260 | 0.568 | 34 | 0.63 | 0.384 | 0.772(0.431,1.383) |
|  |  | A | 198 | 0.432 | 20 | 0.37 | 0.384 | 1.295(0.723,2.318) |
|  | rs7601754 | AA | 175 | 0.764 | 19 | 0.704 | 0.488 | 1.365(0.566,3.292) |
|  |  | AG | 50 | 0.218 | 6 | 0.222 | 0.963 | 0.978(0.374,2.553) |
|  |  | GG | 4 | 0.017 | 2 | 0.074 | 0.066 | 0.222(0.039,1.275) |
|  |  | A | 400 | 0.873 | 44 | 0.815 | 0.231 | 1.567(0.748,3.284) |
|  |  | G | 58 | 0.127 | 10 | 0.185 | 0.231 | 0.638(0.304,1.337) |
|  | rs10174238 | AA | 109 | 0.476 | 14 | 0.5 | 0.81 | 0.908(0.414,1.991) |
|  |  | AG | 102 | 0.445 | 13 | 0.464 | 0.85 | 0.927(0.422,2.036) |
|  |  | GG | 18 | 0.079 | 1 | 0.036 | 0.413 | 2.303(0.296,17.948) |
|  |  | A | 320 | 0.699 | 41 | 0.732 | 0.605 | 0.848(0.454,1.584) |
|  |  | G | 138 | 0.301 | 15 | 0.268 | 0.605 | 1.179(0.631,2.2) |
| Hypergammaglobulinemia | rs10931481 | GG | 25 | 0.281 | 49 | 0.293 | 0.833 | 0.941(0.532,1.663) |
|  |  | AG | 38 | 0.427 | 73 | 0.437 | 0.876 | 0.959(0.571,1.613) |
|  |  | AA | 26 | 0.292 | 45 | 0.269 | 0.7 | 1.119(0.632,1.98) |
|  |  | G | 88 | 0.494 | 171 | 0.512 | 0.705 | 0.932(0.648,1.341) |
|  |  | A | 90 | 0.506 | 163 | 0.488 | 0.705 | 1.073(0.746,1.544) |
|  | rs1400656 | AA | 64 | 0.719 | 118 | 0.707 | 0.833 | 1.063(0.601,1.879) |
|  |  | AG | 21 | 0.236 | 47 | 0.281 | 0.433 | 0.788(0.435,1.429) |
|  |  | GG | 4 | 0.045 | 2 | 0.012 | 0.097 | 3.882(0.697,21.625) |
|  |  | A | 149 | 0.837 | 283 | 0.847 | 0.762 | 0.926(0.563,1.522) |
|  |  | G | 29 | 0.163 | 51 | 0.153 | 0.762 | 1.08(0.657,1.776) |
|  | rs10168266 | CC | 69 | 0.775 | 135 | 0.789 | 0.792 | 0.92(0.496,1.708) |
|  |  | CT | 17 | 0.191 | 34 | 0.199 | 0.88 | 0.951(0.497,1.819) |
|  |  | TT | 3 | 0.034 | 2 | 0.012 | 0.22 | 2.948(0.483,17.974) |
|  |  | C | 155 | 0.871 | 304 | 0.889 | 0.543 | 0.842(0.485,1.464) |
|  |  | T | 23 | 0.129 | 38 | 0.111 | 0.543 | 1.187(0.683,2.063) |
|  | rs3821236 | GG | 29 | 0.326 | 56 | 0.335 | 0.878 | 0.958(0.554,1.656) |
|  |  | AG | 43 | 0.483 | 81 | 0.485 | 0.977 | 0.992(0.593,1.661) |
|  |  | AA | 17 | 0.191 | 30 | 0.18 | 0.823 | 1.078(0.557,2.086) |
|  |  | G | 101 | 0.567 | 193 | 0.578 | 0.82 | 0.958(0.664,1.384) |
|  |  | A | 77 | 0.433 | 141 | 0.422 | 0.82 | 1.044(0.723,1.507) |
|  | rs7601754 | AA | 67 | 0.753 | 127 | 0.76 | 0.891 | 0.959(0.527,1.745) |
|  |  | AG | 19 | 0.213 | 37 | 0.222 | 0.882 | 0.954(0.511,1.781) |
|  |  | GG | 3 | 0.034 | 3 | 0.018 | 0.428 | 1.907(0.377,9.65) |
|  |  | A | 153 | 0.86 | 291 | 0.871 | 0.71 | 0.904(0.532,1.537) |
|  |  | G | 25 | 0.14 | 43 | 0.129 | 0.71 | 1.106(0.651,1.879) |
|  | rs10174238 | AA | 42 | 0.472 | 81 | 0.482 | 0.876 | 0.96(0.574,1.606) |
|  |  | AG | 39 | 0.438 | 76 | 0.452 | 0.828 | 0.944(0.563,1.584) |
|  |  | GG | 8 | 0.09 | 11 | 0.065 | 0.477 | 1.41(0.546,3.643) |
|  |  | A | 123 | 0.691 | 238 | 0.708 | 0.683 | 0.921(0.62,1.368) |
|  |  | G | 55 | 0.309 | 98 | 0.292 | 0.683 | 1.086(0.731,1.613) |
| Rheumatoid factor | rs10931481 | GG | 23 | 0.284 | 51 | 0.291 | 0.902 | 0.964(0.538,1.727) |
|  |  | AG | 35 | 0.432 | 76 | 0.434 | 0.974 | 0.991(0.582,1.687) |
|  |  | AA | 23 | 0.284 | 48 | 0.274 | 0.872 | 1.049(0.584,1.885) |
|  |  | G | 81 | 0.5 | 178 | 0.509 | 0.857 | 0.966(0.666,1.402) |
|  |  | A | 81 | 0.5 | 172 | 0.491 | 0.857 | 1.035(0.713,1.502) |
|  | rs1400656 | AA | 57 | 0.704 | 125 | 0.714 | 0.862 | 0.95(0.533,1.695) |
|  |  | AG | 21 | 0.259 | 47 | 0.269 | 0.875 | 0.953(0.524,1.735) |
|  |  | GG | 3 | 0.037 | 3 | 0.017 | 0.328 | 2.205(0.435,11.171) |
|  |  | A | 135 | 0.833 | 297 | 0.849 | 0.659 | 0.892(0.538,1.48) |
|  |  | G | 27 | 0.167 | 53 | 0.151 | 0.659 | 1.121(0.676,1.859) |
|  | rs10168266 | CC | 61 | 0.753 | 143 | 0.799 | 0.405 | 0.768(0.412,1.432) |
|  |  | CT | 17 | 0.21 | 34 | 0.19 | 0.708 | 1.133(0.59,2.175) |
|  |  | TT | 3 | 0.037 | 2 | 0.011 | 0.16 | 3.404(0.558,20.776) |
|  |  | C | 139 | 0.858 | 320 | 0.894 | 0.24 | 0.718(0.412,1.25) |
|  |  | T | 23 | 0.142 | 38 | 0.106 | 0.24 | 1.393(0.8,2.427) |
|  | rs3821236 | GG | 26 | 0.321 | 59 | 0.337 | 0.799 | 0.929(0.53,1.63) |
|  |  | AG | 39 | 0.481 | 85 | 0.486 | 0.95 | 0.983(0.58,1.666) |
|  |  | AA | 16 | 0.198 | 31 | 0.177 | 0.695 | 1.143(0.585,2.236) |
|  |  | G | 91 | 0.562 | 203 | 0.58 | 0.697 | 0.928(0.637,1.352) |
|  |  | A | 71 | 0.438 | 147 | 0.42 | 0.697 | 1.077(0.74,1.569) |
|  | rs7601754 | AA | 61 | 0.753 | 133 | 0.76 | 0.904 | 0.963(0.522,1.777) |
|  |  | AG | 17 | 0.21 | 39 | 0.223 | 0.815 | 0.926(0.487,1.761) |
|  |  | GG | 3 | 0.037 | 3 | 0.017 | 0.328 | 2.205(0.435,11.171) |
|  |  | A | 139 | 0.858 | 305 | 0.871 | 0.678 | 0.892(0.519,1.532) |
|  |  | G | 23 | 0.142 | 45 | 0.129 | 0.678 | 1.122(0.653,1.926) |
|  | rs10174238 | AA | 38 | 0.469 | 85 | 0.483 | 0.837 | 0.946(0.558,1.603) |
|  |  | AG | 36 | 0.444 | 79 | 0.449 | 0.947 | 0.982(0.579,1.668) |
|  |  | GG | 7 | 0.086 | 12 | 0.068 | 0.604 | 1.293(0.489,3.416) |
|  |  | A | 112 | 0.691 | 249 | 0.707 | 0.712 | 0.927(0.618,1.389) |
|  |  | G | 50 | 0.309 | 103 | 0.293 | 0.712 | 1.079(0.72,1.618) |
| Leukopenia | rs10931481 | GG | 16 | 0.281 | 58 | 0.291 | 0.875 | 0.949(0.493,1.824) |
|  |  | AG | 24 | 0.421 | 87 | 0.437 | 0.828 | 0.936(0.516,1.699) |
|  |  | AA | 17 | 0.298 | 54 | 0.271 | 0.689 | 1.141(0.597,2.181) |
|  |  | G | 56 | 0.491 | 203 | 0.51 | 0.723 | 0.927(0.612,1.407) |
|  |  | A | 58 | 0.509 | 195 | 0.49 | 0.723 | 1.078(0.711,1.635) |
|  | rs1400656 | AA | 40 | 0.702 | 142 | 0.714 | 0.862 | 0.944(0.495,1.801) |
|  |  | AG | 15 | 0.263 | 53 | 0.266 | 0.962 | 0.984(0.504,1.919) |
|  |  | GG | 2 | 0.035 | 4 | 0.02 | 0.51 | 1.773(0.316,9.935) |
|  |  | A | 95 | 0.833 | 337 | 0.847 | 0.728 | 0.905(0.515,1.589) |
|  |  | G | 19 | 0.167 | 61 | 0.153 | 0.728 | 1.105(0.629,1.94) |
|  | rs10168266 | CC | 46 | 0.807 | 158 | 0.778 | 0.641 | 1.191(0.57,2.488) |
|  |  | CT | 10 | 0.175 | 41 | 0.202 | 0.656 | 0.841(0.392,1.804) |
|  |  | TT | 1 | 0.018 | 4 | 0.02 | 0.916 | 0.888(0.097,8.109) |
|  |  | C | 102 | 0.895 | 357 | 0.879 | 0.651 | 1.167(0.598,2.277) |
|  |  | T | 12 | 0.105 | 49 | 0.121 | 0.651 | 0.857(0.439,1.673) |
|  | rs3821236 | GG | 19 | 0.333 | 66 | 0.332 | 0.981 | 1.008(0.539,1.882) |
|  |  | AG | 24 | 0.421 | 100 | 0.503 | 0.278 | 0.72(0.397,1.305) |
|  |  | AA | 14 | 0.246 | 33 | 0.166 | 0.17 | 1.638(0.806,3.329) |
|  |  | G | 62 | 0.544 | 232 | 0.583 | 0.457 | 0.853(0.561,1.297) |
|  |  | A | 52 | 0.456 | 166 | 0.417 | 0.457 | 1.172(0.771,1.782) |
|  | rs7601754 | AA | 43 | 0.754 | 151 | 0.759 | 0.945 | 0.976(0.492,1.937) |
|  |  | AG | 12 | 0.211 | 44 | 0.221 | 0.865 | 0.939(0.458,1.929) |
|  |  | GG | 2 | 0.035 | 4 | 0.02 | 0.51 | 1.773(0.316,9.935) |
|  |  | A | 98 | 0.86 | 346 | 0.869 | 0.788 | 0.921(0.503,1.683) |
|  |  | G | 16 | 0.14 | 52 | 0.131 | 0.788 | 1.086(0.594,1.986) |
|  | rs10174238 | AA | 27 | 0.474 | 96 | 0.48 | 0.933 | 0.975(0.541,1.758) |
|  |  | AG | 25 | 0.439 | 90 | 0.45 | 0.879 | 0.955(0.528,1.727) |
|  |  | GG | 5 | 0.088 | 14 | 0.07 | 0.652 | 1.277(0.44,3.711) |
|  |  | A | 79 | 0.693 | 282 | 0.705 | 0.804 | 0.944(0.601,1.485) |
|  |  | G | 35 | 0.307 | 118 | 0.295 | 0.804 | 1.059(0.674,1.664) |
| Hypocomplementemia | rs10931481 | GG | 8 | 0.276 | 66 | 0.291 | 0.868 | 0.929(0.392,2.203) |
|  |  | AG | 12 | 0.414 | 99 | 0.436 | 0.819 | 0.913(0.417,1.999) |
|  |  | AA | 9 | 0.31 | 62 | 0.273 | 0.673 | 1.198(0.517,2.772) |
|  |  | G | 28 | 0.483 | 231 | 0.509 | 0.709 | 0.901(0.521,1.557) |
|  |  | A | 30 | 0.517 | 223 | 0.491 | 0.709 | 1.11(0.642,1.918) |
|  | rs1400656 | AA | 20 | 0.69 | 162 | 0.714 | 0.788 | 0.892(0.386,2.061) |
|  |  | AG | 7 | 0.241 | 61 | 0.269 | 0.754 | 0.866(0.352,2.129) |
|  |  | GG | 2 | 0.069 | 4 | 0.018 | 0.085 | 4.13(0.722,23.616) |
|  |  | A | 47 | 0.81 | 385 | 0.848 | 0.457 | 0.766(0.378,1.549) |
|  |  | G | 11 | 0.19 | 69 | 0.152 | 0.457 | 1.306(0.645,2.642) |
|  | rs10168266 | CC | 22 | 0.759 | 182 | 0.788 | 0.718 | 0.846(0.342,2.096) |
|  |  | CT | 6 | 0.207 | 45 | 0.195 | 0.877 | 1.078(0.415,2.804) |
|  |  | TT | 1 | 0.034 | 4 | 0.017 | 0.526 | 2.027(0.219,18.777) |
|  |  | C | 50 | 0.862 | 409 | 0.885 | 0.605 | 0.81(0.364,1.801) |
|  |  | T | 8 | 0.138 | 53 | 0.115 | 0.605 | 1.235(0.555,2.746) |
|  | rs3821236 | GG | 9 | 0.31 | 76 | 0.335 | 0.792 | 0.894(0.388,2.058) |
|  |  | AG | 14 | 0.483 | 110 | 0.485 | 0.985 | 0.993(0.458,2.151) |
|  |  | AA | 6 | 0.207 | 41 | 0.181 | 0.731 | 1.183(0.453,3.091) |
|  |  | G | 32 | 0.552 | 262 | 0.577 | 0.713 | 0.902(0.52,1.563) |
|  |  | A | 26 | 0.448 | 192 | 0.423 | 0.713 | 1.109(0.64,1.922) |
|  | rs7601754 | AA | 21 | 0.724 | 173 | 0.762 | 0.653 | 0.819(0.343,1.955) |
|  |  | AG | 6 | 0.207 | 50 | 0.22 | 0.87 | 0.923(0.357,2.392) |
|  |  | GG | 2 | 0.069 | 4 | 0.018 | 0.085 | 4.13(0.722,23.616) |
|  |  | A | 48 | 0.828 | 396 | 0.872 | 0.345 | 0.703(0.337,1.466) |
|  |  | G | 10 | 0.172 | 58 | 0.128 | 0.345 | 1.422(0.682,2.966) |
|  | rs10174238 | AA | 15 | 0.517 | 108 | 0.474 | 0.658 | 1.19(0.549,2.58) |
|  |  | AG | 13 | 0.448 | 102 | 0.447 | 0.993 | 1.004(0.461,2.183) |
|  |  | GG | 1 | 0.034 | 18 | 0.079 | 0.389 | 0.417(0.054,3.243) |
|  |  | A | 43 | 0.741 | 318 | 0.697 | 0.49 | 1.244(0.669,2.314) |
|  |  | G | 15 | 0.259 | 138 | 0.303 | 0.49 | 0.804(0.432,1.495) |
| Monoclonal component | rs10931481 | GG | 9 | 0.333 | 65 | 0.284 | 0.592 | 1.262(0.539,2.952) |
|  |  | AG | 12 | 0.444 | 99 | 0.432 | 0.904 | 1.051(0.471,2.345) |
|  |  | AA | 6 | 0.222 | 65 | 0.284 | 0.499 | 0.721(0.278,1.867) |
|  |  | G | 30 | 0.556 | 229 | 0.5 | 0.44 | 1.25(0.709,2.204) |
|  |  | A | 24 | 0.444 | 229 | 0.5 | 0.44 | 0.8(0.454,1.411) |
|  | rs1400656 | AA | 17 | 0.63 | 165 | 0.721 | 0.324 | 0.659(0.287,1.516) |
|  |  | AG | 8 | 0.296 | 60 | 0.262 | 0.703 | 1.186(0.493,2.851) |
|  |  | GG | 2 | 0.074 | 4 | 0.017 | 0.066 | 4.5(0.784,25.817) |
|  |  | A | 42 | 0.778 | 390 | 0.852 | 0.158 | 0.61(0.306,1.218) |
|  |  | G | 12 | 0.222 | 68 | 0.148 | 0.158 | 1.639(0.821,3.271) |
|  | rs10168266 | CC | 21 | 0.778 | 183 | 0.785 | 0.927 | 0.956(0.366,2.497) |
|  |  | CT | 5 | 0.185 | 46 | 0.197 | 0.879 | 0.924(0.332,2.57) |
|  |  | TT | 1 | 0.037 | 4 | 0.017 | 0.477 | 2.202(0.237,20.448) |
|  |  | C | 47 | 0.87 | 412 | 0.884 | 0.766 | 0.88(0.379,2.045) |
|  |  | T | 7 | 0.13 | 54 | 0.116 | 0.766 | 1.136(0.489,2.641) |
|  | rs3821236 | GG | 8 | 0.296 | 77 | 0.336 | 0.677 | 0.831(0.348,1.985) |
|  |  | AG | 13 | 0.481 | 111 | 0.485 | 0.975 | 0.987(0.444,2.193) |
|  |  | AA | 6 | 0.222 | 41 | 0.179 | 0.584 | 1.31(0.498,3.45) |
|  |  | G | 29 | 0.537 | 265 | 0.579 | 0.559 | 0.845(0.48,1.488) |
|  |  | A | 25 | 0.463 | 193 | 0.421 | 0.559 | 1.184(0.672,2.085) |
|  | rs7601754 | AA | 19 | 0.704 | 175 | 0.764 | 0.488 | 0.733(0.304,1.768) |
|  |  | AG | 6 | 0.222 | 50 | 0.218 | 0.963 | 1.023(0.392,2.671) |
|  |  | GG | 2 | 0.074 | 4 | 0.017 | 0.066 | 4.5(0.784,25.817) |
|  |  | A | 44 | 0.815 | 400 | 0.873 | 0.231 | 0.638(0.304,1.337) |
|  |  | G | 10 | 0.185 | 58 | 0.127 | 0.231 | 1.567(0.748,3.284) |
|  | rs10174238 | AA | 14 | 0.519 | 109 | 0.474 | 0.661 | 1.195(0.538,2.655) |
|  |  | AG | 12 | 0.444 | 103 | 0.448 | 0.973 | 0.986(0.442,2.2) |
|  |  | GG | 1 | 0.037 | 18 | 0.078 | 0.439 | 0.453(0.058,3.534) |
|  |  | A | 40 | 0.741 | 321 | 0.698 | 0.514 | 1.237(0.652,2.347) |
|  |  | G | 14 | 0.259 | 139 | 0.302 | 0.514 | 0.808(0.426,1.533) |
